# The effect of enterocin A/P dipeptide on growth performance, glutathione-peroxidase activity, IgA secretion and jejunal morphology in rabbits after experimental methicillin-resistant *Staphylococcus epidermidis* P3Tr2a Infection

**DOI:** 10.1007/s11259-023-10277-w

**Published:** 2023-12-05

**Authors:** Monika Pogány Simonová, Ľubica Chrastinová, Jana Ščerbová, Valentína Focková, Iveta Plachá, Katarína Tokarčíková, Rudolf Žitňan, Andrea Lauková

**Affiliations:** 1 Centre of Biosciences of the Slovak Academy of Sciences, Institute of Animal Physiology, Šoltésovej 4-6, Košice, 04001 Slovakia; 2 Department of Animal Nutrition, National Agricultural and Food Centre, Hlohovecká 2, Nitra-Lužianky, 95141 Slovakia

**Keywords:** Enterocin, Methicillin-resistant *Staphylococcus*, Immunity, Jejunal morphometry, Rabbits

## Abstract

The increasing frequency of methicillin-resistant (MR) staphylococci in humans and animals need special attention for their difficult treatment and zoonotic character, therefore novel antimicrobial compounds on a natural base against antibiotic-resistant bacteria are requested. Currently, bacteriocins/enterocins present a new promising way to overcome this problem, both in prevention and treatment. Therefore, the preventive and medicinal effect of dipeptide enterocin EntA/P was evaluated against MR *Staphylococcus epidermidis* SEP3/Tr2a strain in a rabbit model, testing their influence on growth performance, glutathione-peroxidase (GPx) enzyme activity, phagocytic activity (PA), secretory (s)IgA, and jejunal morphometry (JM). Eighty-eight rabbits (aged 35 days, meat line M91, both sexes) were divided into experimental groups S (SEP3/Tr2a strain; 1.0 × 10^5^ CFU/mL; dose 500µL/animal/day for 7 days, between days 14 and 21 to simulate the pathogen attack), E (EntA/P; 50 µL/animal/day, 25,600 AU/mL in two intervals, for preventive effect between days 0 and 14; for medicinal effect between days 28 and 42), E + S (EntA/P + SEP3/Tr2a; preventive effect; SEP3/Tr2a + EntA/P; medicinal effect) and control group (C; without additives). Higher body weight was recorded in all experimental groups (*p* < 0.001) compared to control data. The negative influence/attack of the SEP3Tra2 strain on the intestinal immunity and environment was reflected as decreased GPx activity, worse JM parameters and higher sIgA concentration in infected rabbits. These results suggest the promising preventive use of EntA/P to improve the immunity and growth of rabbits, as well as its therapeutic potential and protective role against staphylococcal infections in rabbit breeding.

## Introduction

The routine use of antibiotics in agriculture leads to the alarming rise of antibiotic-resistant bacteria in food animals, presenting a potential public health concern and critical economic issue (increased morbidity/mortality rate; Vidovic and Vidovic 2020). Staphylococci are frequently found as commensals of the skin and mucous membranes, but many of them are also opportunistic pathogens, causing local pyogenic and systemic infections - toxemia and septicemia in livestock, mostly in cattle, pigs, poultry and rabbits (Bonvegna et al. 2021; Silva et al. 2022). From coagulase-positive staphylococci (CPS), *Staphylococcus aureus* has special human and veterinary importance, because of its methicillin-resistance (MR) and multidrug-resistance (MDR). Nevertheless, in recent years, the rate of methicillin-resistant coagulase-negative staphylococci (MR-CNS), such as *S. epidermidis, S. haemolyticus* and *S. chromogenes*, has become more numerous and are involved in nosocomial infections and various animal diseases, due to their invasiveness, biofilm-forming ability, toxin production, hemolysins (Igbinosa et al. 2016). The increasing frequency of methicillin-resistant and multidrug-resistant staphylococci (MRS, MDRS) in animals, containing resistance and pathogenicity genes, needs special attention for their difficult treatment and zoonotic character (Vidovic and Vidovic 2020; Silva et al. 2022; Kasela et al. 2023). Therefore, new antimicrobial compounds directed against antibiotic-resistant bacteria are requested. Current research is focusing on alternative approaches: pro-, pre-, syn-, parapro- and postbiotics – including antimicrobial proteins, organic and fatty acids, herbal extracts and essential oils, to overcome this problem, substitute synthetic drugs in therapy and use preventively as natural feed additives in animal production and veterinary medicine (Zamojska et al. 2021). Among different bioactive compounds, bacteriocins may be considered as promising alternative. These ribosomally synthesized antimicrobial peptides with a broad antimicrobial spectrum produced by Gram-negative and Gram-positive bacteria as well, mostly by lactic acid bacteria (LAB), including enterococci (producing bacteriocins named mostly enterocins (Ents); Franz et al. 2007) are commonly used in the food industry as biopreservatives. However, many bacteriocins have been investigated, only nisin and pediocin PA-1 are considered safe by the Food and Drug Administration (FDA) and approved in the food industry (EFSA 2017), with antimicrobial effect against pathogenic/spoilage bacteria, including *Staphylococcus aureus* (Silva et al. 2018; Vera-Santander et al. 2023). Bacteriocins/enterocins application is also increasingly applied in livestock farms to improve the health and productivity of food animals, regarding their biosafety, antimicrobial, antioxidant, and immunomodulatory activities, and low tendency to develop resistance compared to conventional antibiotics (Bemena et al. 2014; Ben Lagha et al. 2017; Vieco-Saiz et al. 2019; Hernández-González et al. 2021; Pogány Simonová et al. 2020). In vitro antimicrobial and antibiofilm effect of bacteriocins (mostly lantibiotics – nisin (Nisaplin®, Aplin and Barret, United Kingdom) and gallidermin (Enzo Life Sci. Corporation USA, MW2069.4), but also Ents) against MRS and MDR bacteria was repeatedly confirmed, also in the animal (murine) model (Al Atya et al. 2016; Mathur et al. 2018; Belguesmia et al. 2021; Benítez-Chao et al. 2021), showing their ability to eliminate/reduce, particularly MR bacteria. Ents have also great potential during their application in animal farms and veterinary medicine with both, preventive and medicinal effects against bacterial infections (Simons et al. 2020). Despite these findings, knowledge regarding in vivo anti-staphylococcal effect against MRS/MDRS in food animals is limited, resp. has not yet been presented and new studies/experiments to expand these data are required. Therefore, this study aimed to test and compare the preventive and medicinal effect of dipeptide Enterocin (Ent) A/P against methicillin-resistant (MR) *Staphylococcus epidermidis* SEP3Tr2a strain in a rabbit model (food animal model) and their influence on growth performance, glutathione-peroxidase enzyme activity, immune response (phagocytic activity, sIgA), and jejunal morphometry was evaluated. The in vivo testing of the medicinal effect of a new EntA/P on the intestinal morphometry is the novelty of this study.

## Materials and methods

### Animal model

A total of 88 rabbits (meat lines M91 and P91, weaned at 35 days, both sexes, equal male-to-female ratio per treatment) were divided into three experimental groups: S, E, S + E and one control group (C), 22 animals in each. The average live weight of rabbits at the start of the experiment was 1091.7 g ± 149.3. Animals were kept in standard cages (type D-KV-72; 0.61 m x 0.34 cm x 0.33 m; Kovobel company, Domažlice, Czech Republic), two rabbits per cage. A cycle of 16 h light and 8 h dark was used throughout the experiment. The temperature and humidity in the building were recorded continuously by a digital thermograph positioned at the same level as the cages. The heating and ventilation systems allowed the building air temperature maintained within 16 ± 4 °C and the relative humidity to about 70 ± 5% throughout the experiment. Data were recorded continuously with a digital thermograph positioned at the same level as the cages. The experiment was performed in co-operation with our colleagues in Nitra (National Agricultural and Food Centre—NAFC). All care and experimental procedures involving animals followed the guidelines stated in the Guide for the Care and Use of Laboratory Animals approved by the Slovakian State Veterinary and Food Administration and the Ethical Committees of both institutions (permission code: SK CH 17,016 and SK U 18,016).

### Preparation of tested substances

The MR *S. epidermidis* SEP3/Tr2a strain was marked by rifampicin to differentiate it from the total staphylococci and prepared as described previously by Strompfová et al. (2006). The EntA/P (previously named EntEK13, produced by the *E. faecium* EK13 strain, deponed to the Czech Collection of Microorganisms, number CCM7319 (Lauková et al. 2006) was prepared according to Mareková et al. (2007). The activity of EntA/P was tested using the agar spot test according to De Vuyst et al. (1996) against the principal indicator strain *E. avium* EA5 (isolated from piglet feces in our laboratory). Doses of additives and their manner of application were decided on the results of our previous in vivo experiment with rabbit-derived bacteriocin-producing strain *E. faecium* EF2019 (CCM7420; Pogány Simonová et al. 2009).

### Experimental design – treatments

As both tested substances are water-soluble, they were applied to the drinking water of rabbits, using nipple drinkers in all cages. Rabbits in group E received the EntA/P at a dose of 50 µL/animal/day, with activity of 25,600 AU/mL in concentration 0.4 g/L. To test and compare the preventive and also the medicinal effect of the EntA/P, it was added in two intervals to rabbits: during the first 14 days (between days 0 and 14) to control the preventive effect, and between days 28 and 42 to evaluate the medicinal effect. Rabbits in group S received only the MR *S. epidermidis* SEP3/Tr2a strain (1.0 × 10^5^ CFU/mL; Pogány Simonová et al. 2021a) at a dose of 500µL/animal/day for 7 days (between days 14 and 21), to simulate the spoilage/pathogen attack in rabbits. Rabbits in the E + S group firstly consumed the Ent A/P for 14 days (between 0 and 14 days), and after it the SEP3Tr2a strain was applied to animals for 7 days, (between 14 and 21 days of the experiment), to test the preventive effect of EntA/P. After a one-week break, at day 28, to rabbits in the E + S group the EntA/P was applied for 14 days (between 28 and 42 days) to detect the medicinal effect of EntA/P. Control rabbits (C) did not receive any additives in their drinking water. The rabbits were fed with a commercial pelleted diet for growing rabbits (KV, Tekro-Nitra, Ltd., Slovakia; Table 1), with free access to drinking water.

**Table 1:** Nutrient content of commercial granulated diet for growing rabbits

Nutrient content	g.kg^-1^ in original feed	g.kg^-1^ in dry matter
Dry matter	886.65	1000
Crude protein	155.35	174.94
Crude fibre	132.37	149.29
Crude fat	20.3	22.89
Ash	90.08	101.6
Starch	238.71	269.22
Acid detergent fibre	151.69	171.08
Neutral detergent fibre	295.1	332.83
Calcium	15.9	17.94
Phosphorus	4.89	5.51
Magnesium	2.57	2.9
Sodium potassium	1.21	1.36
Iron	564.70*	636.88*
Zinc	97.77*	110.27*
Copper	20.50*	23.12*
Metabolizable energy (MJ.kg^-1^)	11.16	11.02

*mg.kg^-1^ of feed

### **Growth performance**

Body weight (BW; g) and feed consumption (g) were measured every week during the experiment; average daily weight gain (ADWG; the difference between the initial and current weight of animals, divided by the number of days that occurred between weights; g/day) and feed conversion ratio (FCR; feed intake divided by weight gain for a period;.g/g) were calculated mathematically. Health status and mortality were recorded daily throughout the whole experiment.

### Blood sampling and testing of glutathione-peroxidase and phagocytic activity

Blood was sampled from the marginal ear vein (*Vena auricularis*) into dry heparinized Eppendorf tubes at days 0, 14, 21 and 42 for analyses (*n* = 8/group). The activity of glutathione-peroxidase (GPx; µkat/L) was determined by the colorimetric method (Spectrophotometer UV-2550 Shimadzu, Japan) using commercial kit Randox RS 504 (Randox Laboratories Ltd., UK).

The phagocytic activity (PA) was measured by a direct microscopic counting procedure, according to the modified test described by Vetvička et al. (1982): 50 µL of MSH particle suspension (ARTIM, Prague, Czech Republic) was mixed with 100 µL of blood in an Eppendorf-type test tube and incubated at 37 °C for 1 h. Blood smears were then prepared and stained by May-Grünwald and Giemsa-Romanowski. PA was calculated as the number of white cells containing at least three engulfed particles per 100 white cells (monocytes/granulocytes).

### Slaughtering, intestinal IgA and morphometry testing

At days 21 and 42, rabbits were randomly selected for slaughter (*n* = 8), stunned with electronarcosis (50 Hz, 0.3 A/rabbit/4s), immediately hung by the hind legs on the processing line and quickly bled by cutting jugular veins and carotid arteries. The concentration of immunoglobulin A (IgA) in the intestinal wall was measured using the competitive inhibition enzyme immunoassay technique (Rabbit Immunoglobulin A, IgA ELISA kit, Cusabio, Houston, TX, USA). Samples of the intestinal wall were prepared according to Nikawa et al. (1999) and analyzed using the Multireader Synergy HTX (Biotek USA), at the wavelength 450 nm according to the manufacturer of the Cusabio kit.

To test morphometry (villus cut surface, villus circumference, villus height, crypt depth and villus height:crypt depth ratio), intestinal tissue (1 cm^2^) of proximal jejeunum was sampled and treated as previously described by Žitňan et al. (2008). Briefly, intestinal tissue (1 cm^2^) from the proximal jejunum was fixed in a 4% neutral formaldehyde solution. After being rinsed in water, samples were dehydrated in a graded series of ethanol (30%, 50%, 70%, 90%, and absolute ethanol), cleared in benzene and embedded in paraffin. Sections of 5 μm thickness (10 slices of each sample) were stained with hematoxylin/eosin and observed under a light microscope. The height, circumference, and cut surface area of 30 villi and depth of 30 crypts were determined by the computer-operated *Image C* picture analysis system (Imtronic GmbH, Berlin, Germany) and the interactive measurement (IMES) analysis program, by using a color video camera (SONY 3 CCD, Sony Electronics Ltd., Tokyo, Japan) and a light microscope (Axiolab, Carl Zeiss AG, Jena, Germany).

### Statistical analysis

Treatment effects on tested parameters were analyzed using two-way ANOVA, followed by a Bonferroni post-hoc test for pair‐wise comparisons, where appropriate. The statistical model included the time, treatment effects and their interaction. All statistical analyses were performed by the GraphPad Prism statistical software (GraphPad Prism version 6.0, GraphPad Software, San Diego, CA, USA). Differences between the mean values of the different dietary treatments were considered statistically significant at *p* < 0.05. Data are expressed as means and standard deviations of the mean (SD).

## Results

The animals were in good health throughout the experiment. All tested zootechnical parameters were influenced by time (BW, FCR), treatment (BW, ADWG) and their interaction (ADWG; Table 2). Higher BW was recorded in all experimental groups (*p* < 0.001) compared to control data. The ADWG was affected by both additives, but mostly the EntA/P (days 0–14; E, S + E vs. C: *p* < 0.001; S vs. C: *p* < 0.01; days 14–21: E vs. C, S: *p* < 0.05).

**Table 2:** The effect of methicillin-resistant *S. epidermidis* SEP3/Tr2a (S), EntA/P (E) and their combinative application (S + E) on growth performance, phagocytic and glutathione-peroxidase activity, and intestinal IgA concentration of rabbits

Parameter	Day	S	E	S + E	C	*p*-value		
						Time	Treatment	Interaction
Body weight (g)	0	1140.20 ± 149.30	1152.90 ± 178.20	1060.90 ± 150.70	1012.90 ± 118.80	<0.0001	< 0.0001	0.3759
	14	1734.50 ± 152.30^a^	1735.00 ± 260.00^a^	1691.50 ± 213.10^a^	1487.80 ± 203.90^b^			
	21	2014.80 ± 186.10^a^	2052.70 ± 266.10^a^	1999.00 ± 212.20^a^	1759.70 ± 218.60^b^			
	42	2711.80 ± 243.90^a^	2854.10 ± 330.70^a^	2696.70 ± 272.30^a^	2469.20 ± 285.50^b^			
Average daily weight gain	0–14	42.45 ± 8.67^a^	41.58 ± 5.85^a^	45.04 ± 9.54^a^	33.92 ± 8.53^b^	<0.0001	< 0.0001	0.0049
(ADWG; g/day/rabbit)	14–21	40.04 ± 8.82^a^	45.39 ± 8.13^b^	43.93 ± 7.80^ab^	38.85 ± 10.61^a^			
	28–42	33.19 ± 4.75	38.16 ± 3.05	33.22 ± 4.18	33.79 ± 3.19			
Feed conversion ratio	0–21	2.78 ± 0.92	2.37 ± 0.44	2.60 ± 0.67	2.64 ± 0.20	<0.0001	0.1207	0.1540
(FCR; g/g)	21–42	4.26 ± 0.75^ab^	4.17 ± 0.89^ab^	4.38 ± 0.54^a^	3.90 ± 0.38^b^			

^a,b,c,d^Mean values within lines with different superscript letters are significantly different (*p* < 0.05) using the Bonferroni post-test

S - *S. epidermidis* SEP3/Tr2a, E – EntA/P, S + E - *S. epidermidis* SEP3/Tr2a + EntA/P, C – control, ADWG – average daily weight gain, FCR – feed conversion ratio

**Fig. 1:**
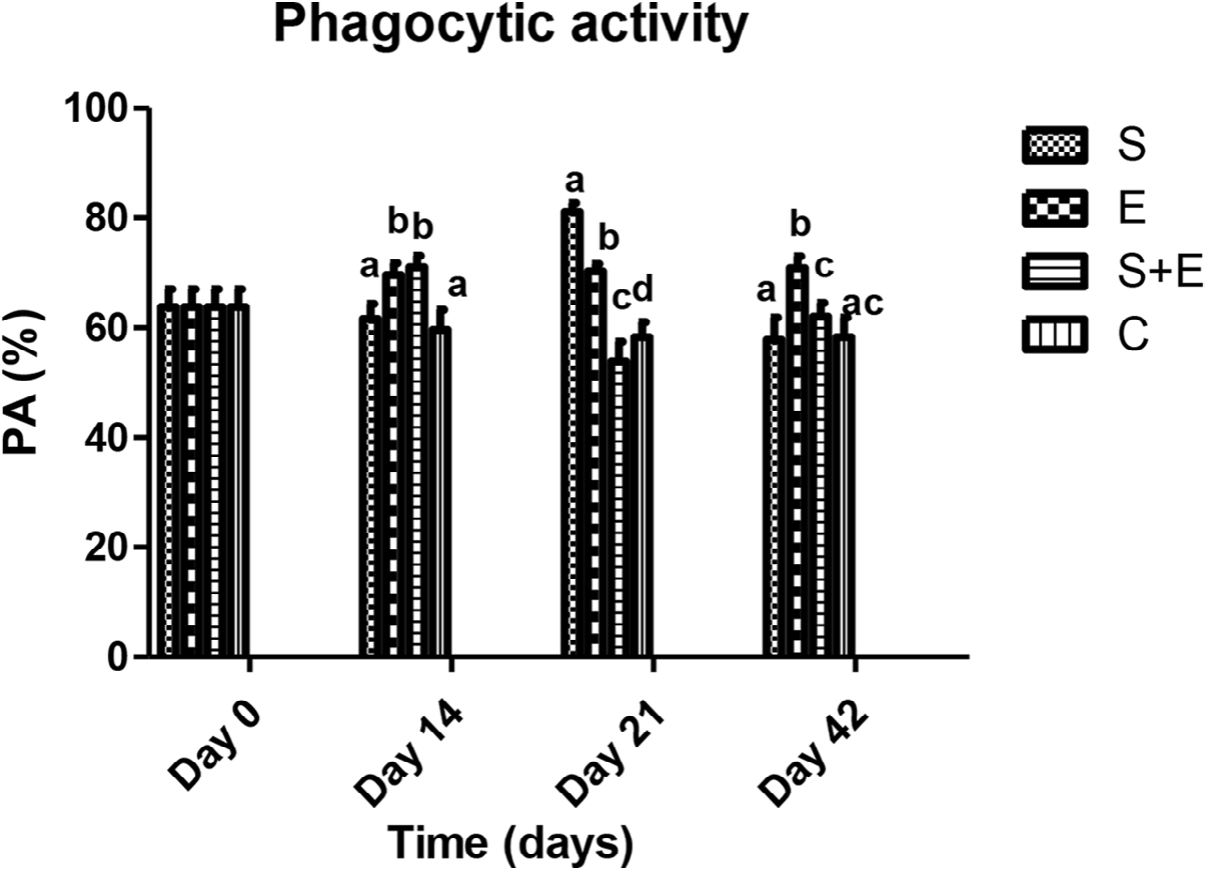
The effect of methicillin-resistant *S. epidermidis* SEP3/Tr2a, EntA/P and their combinative application on phagocytic activity using a direct microscopic counting procedure, according to the modified test of Vetvička et al. (1982). S - *S. epidermidis* SEP3/Tr2a, E – EntA/P, S + E - *S. epidermidis* SEP3/Tr2a + EntA/P, C – control. ^a,b,c,d^ Mean values within lines with different superscript letters are significantly different (*p* < 0.05) using the Bonferroni post-test

**Fig. 2:**
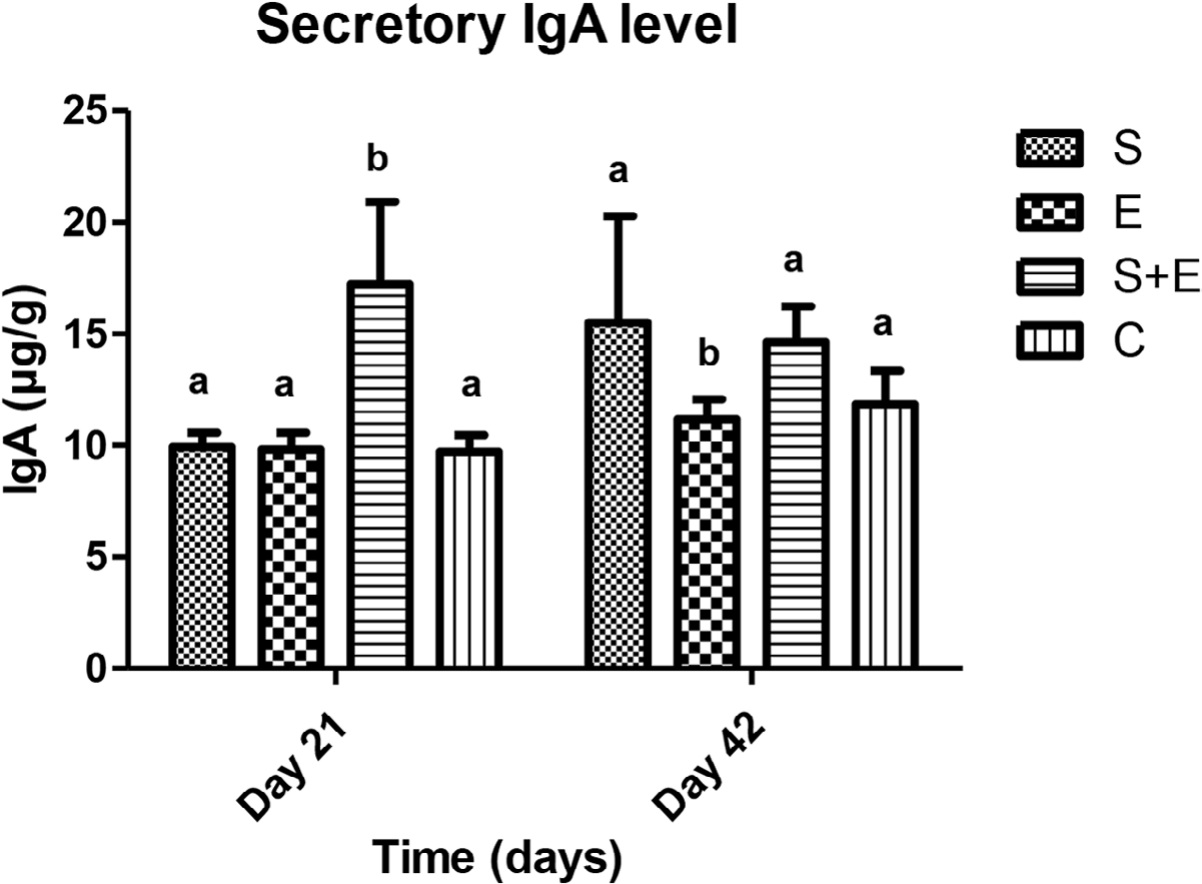
The effect of methicillin-resistant *S. epidermidis* SEP3/Tr2a, EntA/P and their combinative application on secretory IgA production, prepared and measured using the competitive inhibition enzyme immunoassay technique. S - *S. epidermidis* SEP3/Tr2a, E – EntA/P, S + E - *S. epidermidis* SEP3/Tr2a + EntA/P, C – control. Hb – hemoglobin; ^a,b,^Mean values within lines with different superscript letters are significantly different (*p* < 0.05) using the Bonferroni post-test

**Fig. 3:**
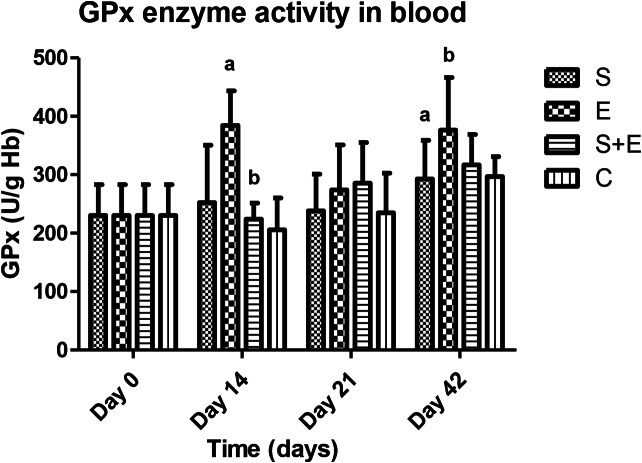
The effect of methicillin-resistant *S. epidermidis* SEP3/Tr2a, EntA/P and their combinative application on glutathione-peroxidase activity determined by colorimetric method. S - *S. epidermidis* SEP3/Tr2a, E – EntA/P, S + E - *S. epidermidis* SEP3/Tr2a + EntA/P, C – control. ^a,b,c,d^ Mean values within lines with different superscript letters are significantly different (*p* < 0.05) using the Bonferroni post-test

While PA (days 14, 21, 42; E vs. S, E + S, C: *p* < 0.001; Fig. 1) and sIgA levels (day 21; S + E vs. S, E, C: *p* < 0.001; day 42; E vs. S, S + E: *p* < 0.001; S, S + E vs. C: *p* < 0.05; Fig. 2) were influenced by time, treatment and their interaction, the blood GPx enzyme activity was affected by time and treatment (day 14; S vs., S + E: *p* < 0.001; day 42; S vs. E: *p* < 0.05; Fig. 3).

**Fig. 4:**
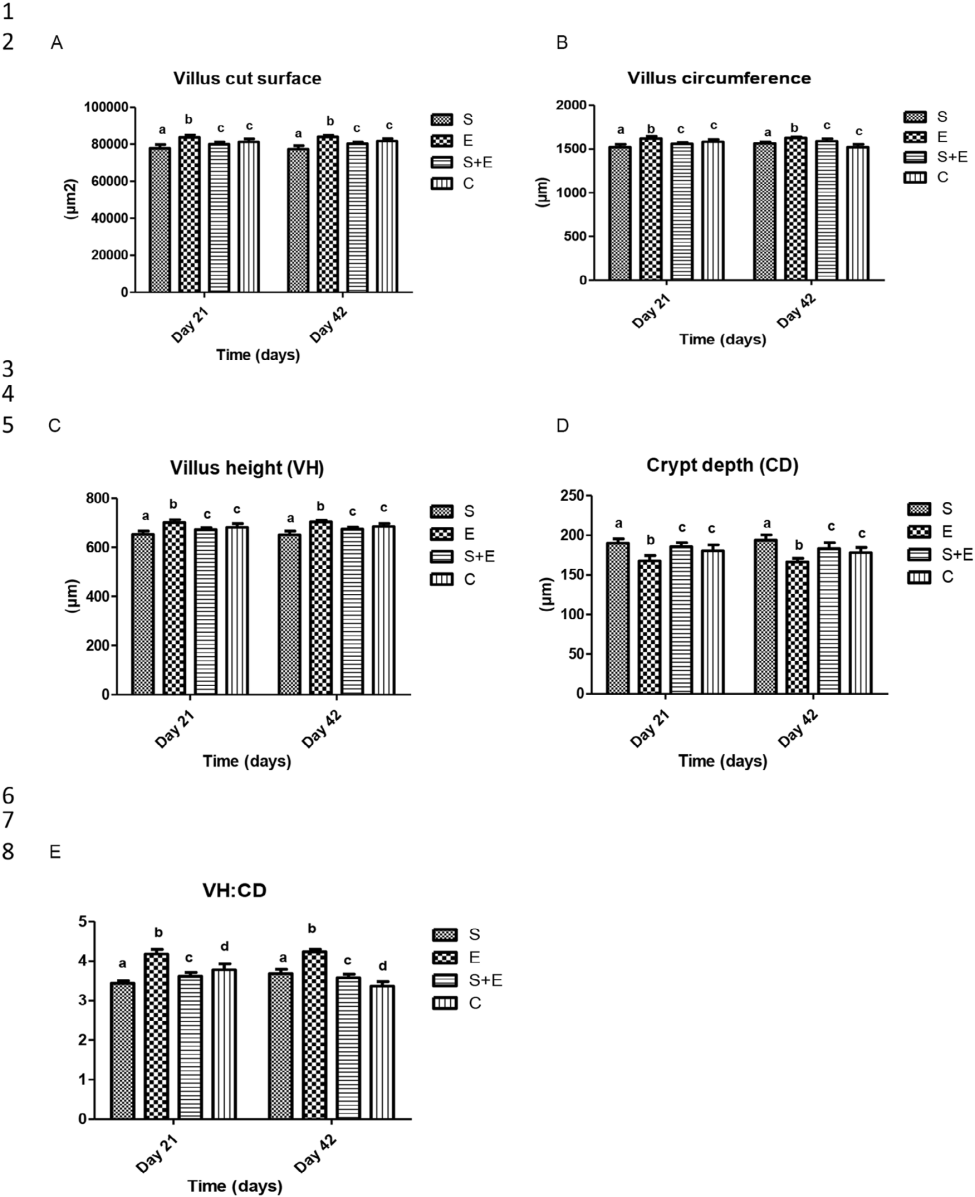
The effect of methicillin-resistant *S. epidermidis* SEP3/Tr2a, EntA/P and their combinative application on jejunal morphometry parameters tested according to Žitňan et al. (2008) – villus cut surface **A**, villus circumference **B**, villus height **C**, crypt depth **D**, villus height and crypt depth ratio (VH:CD; E). S - *S. epidermidis* SEP3/Tr2a, E – EntA/P, S + E - *S. epidermidis* SEP3/Tr2a + EntA/P, C – control. ^a,b,c,d^Mean values within lines with different superscript letters are significantly different (*p* < 0.05) using the Bonferroni post-test

Morphometry parameters were influenced by treatment (Table 2). The lowest values of all tested parameters were measured after the MR SEP3/Tr2a strain, which reflects the negative effect of the applied strain on the intestinal epithelium and environment. The opposite results were observed in the case of the EntA/P administration to rabbits, showing the tendency to improve the jejunal morphological parameters till to the end of the experiment (day 42), with a significant increase in resp. the highest level of all tested parameters (*p* < 0.01) compared to control and other experimental groups. Optimized values were noted testing the medicinal effect of EntA/P after the MR SEP3/Tr2a strain application (day 42) (see Fig. 4).

## Discussion

Weaning is a key period associated with stress, during which rabbits are susceptible to dietary changes and infections, which have adverse impacts on their health and production. The most rapid and significant changes are seen in the growth of rabbits, such as lack of appetite, stunting, and lower gains. However, we suspected lower weight gains in rabbits after the application of the potential pathogenic MR SEP3Tr2a strain, no negative effect on the growth performance of rabbits was noted. Similar results – higher weight gain – were obtained also after the application of a potentially pathogenic biofilm-forming *Enterococcus hirae* Kr8^+^ strain to rabbits (Lauková et al. 2022). We can speculate that applied strains may affect the intestinal environment and health, but in the gut is a complex interaction of the microbiome, enzymes, epithelial barrier and immunity, which may mitigate the pathogenicity resp. the negative effect of a potential pathogenic strain. The stimulatory effect of EntA/P application on animals´ growth and weight gains was noted, in accordance with our previous results achieved after several enterocins (Ent4231, Ent7420, EntM, durancin, etc.) application to rabbits (Pogány Simonová et al. 2015, 2021b). These findings repeatedly confirm the prosperity of enterocins as feed additives with prophylactic character to improve animal production. Moreover, optimized ADWG values noted in experimentally infected rabbits with the MR SEP3/Tr2a strain receiving EntA/P after the infection underline the medicinal effect of EntA/P.

Dietary changes, manipulation and infections can induce a stress response in animals with immoderate production of reactive oxygen species (ROS), which is controlled by the antioxidant system of the host organism. Monitoring the GPx activity in blood resp. in neutrophils is one of the markers of the antioxidant defensive system and stress reaction. Chakraborty et al. (2012) present the susceptibility of neutrophils to *S. aureus* infection through the increased production of nitric oxide, which leads to decreased antioxidant status, similar to our findings – lower level of GPx enzyme after MR SEP3Tr2a strain application. It is known, that lactic acid bacteria (LAB), which are usually producer strains of bacteriocins have antioxidant properties. However, the mechanisms underlying the antioxidant activity of probiotics/beneficial bacteria/LAB are not completely understood; however, it has been suggested that LAB may play antioxidant roles through scavenging ROS, chelating metals, increasing antioxidant enzymes levels, and modulating the microbiota (Feng and Wang 2020). Kim et al. (2022) also documented the ability of (LAB) and their metabolites–bacteriocins to remove the ROS and maintain the intestinal oxidation–reduction balance, confirming their antioxidant activity. When LAB are attached to the intestinal lumen, their metabolites – bacteriocins increase to remove ROS, thereby maintaining the intestinal oxidation–reduction balance. Xin et al. (2014) also presented alleviated high-fat diet-induced oxidative stress and changed intestinal Firmicutes/Bacteroidetes ratio in mice after *Lactobacillus johnsonii* BS15 supplementation. The gut microbiota modulation by LAB and their bacteriocins can improve the host redox state and protect the neutrophils from such infection by decreasing nitrite-oxide (NO) generation, lipid, and protein damage and also by increasing the antioxidant status. Significantly increased GPx activity in rabbits receiving the EntA/P alone (E) and after the MR SEP3/Tr2a application (S + E) also confirming previous findings and the potential use of EntA/P as free radical scavenger with both, preventive and medicinal effects against staphylococcal infection in rabbit breeding. Of course, further experiments are required to verify this hypothesis.

Several studies present also the immunostimulatory effect of postbiotics – bacteriocins (Benítez-Chao et al. 2021. The immunomodulatory effect of tested bacteriocins is expressed as increased CD4 + and CD8 + T lymphocyte proliferation and cytokine IL-6 production (Hernández-González et al. 2021). Bacteriocins/enterocins can also stimulate phagocytic activity (PA), as a parameter of non-specific immunity. Enterocin therapy prevented the suppression of phagocytosis of peripheral blood mononuclear cells caused by *Trichinella spiralis* infection in the intestinal phase of trichinellosis and stimulated PA and oxidative burst during the migration of new-born larvae into muscles (Vargová et al. 2023). In vivo PA stimulation was noted in rabbits and horses after their dietary bacteriocins/enterocins supplementation (Lauková et al. 2018, 2022; Pogány Simonová et al. 2013, 2022). The immunostimulatory effect of EntA/P (E) was recorded during the whole experiment, similar to previous results. Optimized PA level in the S + E group after EntA/P addition to experimentally infected rabbits (day 42) has shown the immunomodulatory resp. medicinal effect of the tested dipeptide. The MR SEP3/Tr2a strain application also stimulated the PA in infected rabbits (S; day 21), but surprisingly, decreased PA was noted in S + E group (day 21), compared to other groups. Probiotics/beneficial bacteria and postbiotics can enhance the immune response of rabbits through the modulation of gut microbiota, by supporting/improving gut-associated lymphoid tissues and stimulating the IgA system (Zamojska et al. 2021). Slight increase of sIgA levels in rabbits after EntA/P supplementation can confirm this finding, i.e. to improve the intestinal health and modulate the immune response of the host organism. On the other hand, a more significant increase of sIgA level noted immediately after the MR SE strain application in the S + E group (day 21) suggested the negative attack of the potentially pathogenic MR strain on the intestinal epithelium/lymphoid tissue resp. immunity. Optimization (decrease) of high sIgA level after 2 weeks of EntA/P medicinal application (day 42 vs. day 21) underlines the protective effect of tested EntA/P against the pathological attack in the intestine.

The complex of intestinal epithelium, microbiome and immunity forms a balanced/stable gut environment and homeostasis. Dietary dysbiosis, metabolic changes and the presence of gastrointestinal infection agents – bacteria, viruses, and parasites can influence this stability, which directly disrupt gut integrity (Berkes et al. 2003). Impaired morphometric parameters in this study reflect the negative influence of resp. attack of the MR SEP3/Tr2a strain on rabbits´ intestine. These changes are closely related to intestinal microbiota composition, metabolic activities and immunity. Outgoing from decreased villus surface area, circumference and VH:CD ratio values, we expected lower nutrient resorption and weight gains in experimentally infected rabbits, but their higher body weight compared to control animals did not confirm this assumption. Therefore, we hypothesize stronger resp. more direct effect of the MR SEP3/Tr2a strain on gut lymphoid tissue, than on gastrointestinal microbiota or metabolism. Stimulated secretion of sIgA as a local defense mechanism can confirm this hypothesis, but more detailed studies about intestinal epithelial integrity and immunity are needed to extend knowledge regarding the complexity of microbiological, physiological and immunological processes in the gut. The beneficial influence of dietary probiotic and postbiotic supplementation on intestinal morphological parameters and their improvement of rabbits was reported in several previous studies (Oso et al. 2013; Pogány Simonová et al. 2015, 2020, 2022), similar to present results achieved after the EntA/P application to rabbits with preventive goal. Improved morphometry parameters suggest better intestinal functionality, strengthening of the epithelium, preventing the entry and gut colonization of pathogenic bacteria, increasing of commensal bacterial activities, mucosal immunity (IgA production), and nutrient absorption (enlargement of surface area), leading also to better health status and higher weight gains in animals (Aggarwal et al. 2022; Zhong et al. 2022). The EntA/P applied to experimentally infected rabbits before the infection was able to strengthen the jejunal epithelium, and thus prevent significant damage to the intestinal barrier, compared to untreated infected rabbits. While there are several studies on the preventive administration of postbiotics/bacteriocins in animals (Aggarwal et al. 2022; Zhong et al. 2022), studies on their in vivo therapeutic effect against infections and intestinal homeostasis are still scarce. Therefore, the novelty of this study is testing the therapeutic effect of EntA/P against staphylococcal infection in rabbits directly in the gut, through monitoring morphological changes. The optimized values of morphological parameters in rabbits receiving EntA/P after the MR SEP3/Tr2a strain infection testify to the medicinal effect of EntA/P. To the best of our current knowledge, these results are the first to present the in vivo therapeutic action of enterocins in general on jejunal morphometry.

## Conclusion

The experimental application of potentially pathogenic MR SEP3Tr2a strain did not negatively influence the growth of rabbits, but significantly impaired the antioxidant and immune response of infected animals. The prophylactic application of EntA/P to untreated rabbits (without MRSEP3Tr2a) was reflected in their higher weight gains and lower feed conversion. Moreover, improved jejunal morphometry and enhanced immunity and antioxidant defense system were noted. Optimized/improved values of growth, morphometry and immunity parameters in experimentally infected rabbits receiving EntA/P after their infection underline the medicinal effect of the EntA/P. Prophylactic administration of EntA/P to rabbits before their infection enhanced the animal´s health against the MR SEP3/Tr2a strain. These results suggest the promising use of dipeptide EntA/P as a feed additive and potential therapeutic agent against staphylococcal infection in rabbits, to improve their productivity, health status and immunity.

## Data Availability

All data generated or analyzed during this study are included in this article.

## Abbreviations

MR: Methicillin-resistant
Ent: Enterocin
SE: *Staphylococcus epidermidis*
GPx: Glutathione-peroxidase
PA: Phagocytic activity
sIgA: Secretory immunoglobulin A
JM: Jejunal morphometry
CPS: Coagulase-positive staphylococci
MDR: Multidrug-resistance
MR-CNS: Methicillin-resistant coagulase-negative staphylococci
MRS: Methicillin-resistant staphylococci
MDRS: Multidrug-resistant staphylococci
LAB: Lactic acid bacteria
NAFC: National Agricultural and Food Centre
CCM: Czech Culture Collection of Microorganisms
BW: Body weight
ADWG: average daily weight gain
FCR: feed conversion ratio
NO: Nitrite-oxide
VH: Villus height
CD: Crypt depth
