# Correction to: The effect of enterocin A/P dipeptide on growth performance, glutathione-peroxidase activity, IgA secretion and jejunal morphology in rabbits after experimental methicillin-resistant *Staphylococcus epidermidis* P3Tr2a infection

**DOI:** 10.1007/s11259-023-10284-x

**Published:** 2023-12-19

**Authors:** Monika Pogány Simonová, Ľubica Chrastinová, Jana Ščerbová, Valentína Focková, Iveta Plachá, Katarína Tokarčíková, Rudolf Žitňan, Andrea Lauková

**Affiliations:** 1 Centre of Biosciences of the Slovak Academy of Sciences, Institute of Animal Physiology, Šoltésovej 4-6, Košice, 04001 Slovakia; 2 Department of Animal Nutrition, National Agricultural and Food Centre, Hlohovecká 2, Nitra-Lužianky, 95141 Slovakia

**Correction to: Veterinary Research Communications**

10.1007/s11259-023-10277-w.

In the original online version of this article, data of Time effect are included in Table 2, but missing in the PDF version.

The original article has been corrected.

